# Association of inflammatory markers with abnormal liver biochemistry in hospitalized patients with COVID-19: a cross-sectional study

**DOI:** 10.3389/fpubh.2026.1892718

**Published:** 2026-09-21

**Authors:** Omar Al-Omair

**Affiliations:** College of Medicine, King Faisal University, Al-Ahsa, Saudi Arabia

**Keywords:** abnormal liver biochemistry, COVID-19, ferritin, hospitalized patients, Inflammatory markers

## Abstract

**Background:**

Coronavirus disease 2019 (COVID-19) is frequently accompanied by extrapulmonary manifestations, including abnormal liver biochemistry. Although inflammatory markers are widely used in clinical assessment, their associations with hepatic biochemical abnormalities in hospitalized patients with COVID-19 remain incompletely defined.

**Methods:**

This retrospective cross-sectional secondary analysis included adults admitted with RT-PCR-confirmed COVID-19 at King Fahad Hospital Hofuf, Al-Ahsa, Saudi Arabia, from August through October 2020. Abnormal liver biochemistry was defined as alanine aminotransferase (ALT) >40 U/L, aspartate aminotransferase (AST) >40 U/L, or total bilirubin >21 μmol/L. C-reactive protein (CRP), ferritin, and D-dimer were evaluated as exposure variables. Group comparisons used nonparametric tests, correlations were assessed using Spearman coefficients, and marker-specific multivariable logistic regression models adjusted for age, sex, diabetes mellitus, hypertension, and chronic kidney disease.

**Results:**

Among 631 hospitalized patients (mean age 54.4 ± 14.4 years; 64.1% male), abnormal liver biochemistry was present in 59.2%. Ferritin levels were significantly higher in patients with abnormal than normal liver biochemistry (*p* < 0.001), whereas CRP and D-dimer did not differ significantly. Ferritin correlated positively with ALT, AST, and total bilirubin. In adjusted analysis, ferritin remained associated with abnormal liver biochemistry (adjusted odds ratio 3.59 per ten-fold increase; 95% CI 2.19–5.90; *p* < 0.001). CRP and D-dimer were not independently associated; these null findings should be interpreted cautiously because these markers had substantially more missing data.

**Conclusions:**

Abnormal liver biochemistry was common in this cohort of hospitalized patients with COVID-19. Ferritin showed the most consistent association with hepatic biochemical abnormalities, supporting its potential value as a marker of inflammation-related liver involvement in this population. Prospective studies with standardized laboratory assessment are needed to confirm temporality and clinical utility.

## Introduction

Coronavirus disease 2019 (COVID-19), caused by severe acute respiratory syndrome coronavirus 2 (SARS-CoV-2), is a systemic illness with clinically important extrapulmonary manifestations (1, 2). Hepatic involvement most often presents as abnormal liver biochemistry rather than overt liver failure and is common among hospitalized patients (3).

Elevations in alanine aminotransferase (ALT), aspartate aminotransferase (AST), and bilirubin have been reported across hospitalized COVID-19 cohorts, although prevalence varies with disease severity, case mix, sampling time, and the thresholds used (4–6). Most abnormalities are mild or moderate, but higher aminotransferase levels have been associated with intensive care use, longer hospitalization, and mortality (5, 6). Identifying factors associated with abnormal liver biochemistry may therefore support clinical monitoring and risk assessment.

Hepatic involvement in COVID-19 is likely multifactorial. Proposed pathways include direct viral effects on hepatocytes and cholangiocytes, hypoxic or ischemic injury during respiratory failure, drug-induced liver injury, exacerbation of pre-existing liver disease, and immune-mediated or endothelial injury (7–9). The relative contribution of these mechanisms may differ between patients and across phases of illness.

Severe COVID-19 can be accompanied by dysregulated inflammatory signaling and elevations in acute-phase, coagulation, and tissue-injury biomarkers (10–12). C-reactive protein (CRP) reflects interleukin-6-driven hepatic acute-phase synthesis, ferritin reflects iron homeostasis as well as macrophage and inflammatory activation, and D-dimer reflects fibrin formation and degradation in COVID-19-associated coagulopathy (11, 12).

Ferritin is of particular interest because hyperferritinemia is associated with severe COVID-19 and may reflect both systemic inflammation and perturbed iron regulation (11, 13). In hospitalized cohorts, ferritin has correlated with aminotransferase elevation and broader inflammatory activity (13, 14). Mechanistically, ferritin may be linked to cytokine activation, macrophage responses, oxidative stress, and altered hepatic iron handling (15–18). CRP and D-dimer may also accompany liver biochemical abnormalities, although their relationships are not necessarily liver-specific (14, 19).

Studies examining inflammatory markers alongside liver tests have produced heterogeneous results, partly because of differences in design, disease severity, measurement timing, outcome definitions, and handling of missing laboratory data (13, 14, 20, 21). Saudi hospital cohorts have documented frequent hepatobiliary and biochemical abnormalities, but focused analyses of inflammatory markers in relation to liver biochemistry remain limited (22, 23).

This cross-sectional study aimed to examine the relationship between CRP, ferritin, and D-dimer and abnormal liver biochemistry among hospitalized patients with COVID-19. By evaluating unadjusted associations and adjusted regression models, this study sought to identify inflammatory markers associated with hepatic biochemical abnormalities while acknowledging the limitations of cross-sectional observational data.

## Methods

### Study design and reporting standards

This study was a retrospective, hospital-based, cross-sectional secondary analysis of routinely collected clinical data. The parent cohort was previously analyzed for factors associated with length of hospital stay and in-hospital mortality (23); the present analysis addresses a distinct question concerning inflammatory markers and abnormal liver biochemistry. The parent report required complete records and a hospital stay longer than one day, whereas the present analysis required at least one available liver biochemistry parameter; accordingly, the analytic cohort comprised 631 patients, compared with 630 in the parent report. The study is reported in accordance with the Strengthening the Reporting of Observational Studies in Epidemiology (STROBE) guidance for cross-sectional studies.

### Study setting and population

The study was conducted at King Fahad Hospital Hofuf, a referral hospital in Al-Ahsa, Saudi Arabia. Records covered consecutive eligible admissions from August through October 2020, during the first pandemic wave. This period preceded COVID-19 vaccination and most later variants and treatment protocols; it therefore provides important temporal context and limits direct generalization to contemporary care.

### Eligibility criteria

Patients were eligible for inclusion if they met the following criteria:

- Age ≥18 years;
- Laboratory-confirmed SARS-CoV-2 infection by RT-PCR;
- Hospital admission for COVID-19-related illness;
- Availability of at least one liver biochemistry parameter (ALT, AST, or total bilirubin) during hospitalization.

Patients with documented chronic liver disease were not excluded a priori because the objective was to evaluate abnormal liver biochemistry in a real-world hospitalized COVID-19 cohort. Pre-existing liver disease was identified from the medical record and described as a baseline characteristic where available. Because documentation was incomplete, chronic liver disease was not included in the primary multivariable model and should therefore be considered a potential source of residual confounding.

### Data collection and variables

Data were retrospectively extracted from electronic medical records using a standardized data abstraction form. Collected variables included age, sex, clinical comorbidities, laboratory parameters obtained during hospitalization, and clinical outcome indicators recorded in hospital records.

All laboratory values were obtained as part of routine clinical care. For patients with multiple laboratory measurements during hospitalization, the first available value recorded after admission was used for analysis to reduce bias related to disease progression or treatment effects.

### Inflammatory markers

The primary exposure variables were CRP, ferritin, and D-dimer. These markers were selected because they reflect systemic inflammation, immune dysregulation, and COVID-19-associated coagulopathy. Values were analyzed as continuous variables and log10-transformed in regression analyses because their distributions were right-skewed. Laboratory results are reported as CRP (mg/L), ferritin (ng/mL), and D-dimer (mg/L fibrinogen-equivalent units [FEU]).

### Liver biochemistry and outcome definition

The primary outcome was abnormal liver biochemistry, defined using the following thresholds:

- ALT >40 U/L;
- AST >40 U/L;
- Total bilirubin >21 μmol/L.

A composite binary outcome was constructed whereby patients were classified as having abnormal liver biochemistry if any of the above criteria were met. This composite definition was chosen to capture common hepatocellular and bilirubin abnormalities in routine clinical data. Because AST is not liver-specific and may also reflect muscle injury or systemic illness, the outcome should be interpreted as abnormal liver biochemistry rather than definitive liver-specific injury.

### Covariates and potential confounders

Potential confounding variables were selected a priori based on biological plausibility and previous literature. The primary adjusted model included age, sex, diabetes mellitus, hypertension, and chronic kidney disease. Other potentially important variables, including COVID-19 severity, intensive care admission, oxygen requirement, body mass index, hepatotoxic medications, antiviral or steroid treatment, alcohol use, viral hepatitis, and baseline liver biochemistry, were not consistently available in the study dataset and were therefore considered potential sources of residual confounding.

### Handling of missing data

Missing data were assessed for all variables. Liver biochemistry parameters demonstrated low missingness, whereas inflammatory markers, particularly CRP and D-dimer, had substantial missingness because laboratory testing was guided by clinical judgment. Analyses were conducted using an available-case approach, whereby patients with missing values for a given marker were excluded only from analyses involving that marker. No imputation was performed for inflammatory markers to avoid introducing additional bias related to non-random test ordering. Available denominators are reported in the tables, and the potential impact of selective testing is addressed in the limitations (Table 1).

**Table 1:** Availability of key laboratory variables.

Variable	Patients with data, *n/N* (%)	Missing, *n/N* (%)
ALT	615/631 (97.5)	16/631 (2.5)
AST	617/631 (97.8)	14/631 (2.2)
Total bilirubin	615/631 (97.5)	16/631 (2.5)
Composite abnormal liver biochemistry	618/631 (97.9)	13/631 (2.1)
CRP	107/631 (17.0)	524/631 (83.0)
Ferritin	401/631 (63.5)	230/631 (36.5)
D-dimer	109/631 (17.3)	522/631 (82.7)

The full cohort denominator was *N* = 631. The denominator for each row is shown explicitly; percentages may differ slightly because of rounding.

### Statistical analysis

All statistical analyses were performed using IBM (International Business Machines Corporation Statistics (version 28) 1 New Orchard Road, Armonk, New York, United States) and Python-based statistical packages. Continuous variables were assessed for normality using visual inspection of histograms and the Shapiro-Wilk test. Normally distributed variables were summarized as mean and standard deviation (SD), while non-normally distributed variables were summarized as median and interquartile range (IQR). Categorical variables were reported as frequencies and percentages using available denominators.

Comparisons between patients with and without abnormal liver biochemistry were performed using Mann–Whitney *U* tests for continuous non-parametric variables and chi-square or Fisher exact tests for categorical variables, as appropriate. Associations between inflammatory markers and continuous liver parameters were assessed using Spearman rank correlation coefficients.

Multivariable logistic regression models were constructed with abnormal liver biochemistry (yes/no) as the dependent variable. Separate models were fitted for CRP, ferritin, and D-dimer because data availability differed between markers. Inflammatory markers were entered as log10-transformed continuous variables; adjusted odds ratios therefore represent the change in odds associated with a ten-fold increase in the marker. Results are presented as adjusted odds ratios (aORs) with 95% confidence intervals (CIs). Model assumptions were assessed, including absence of multicollinearity and linearity of continuous predictors on the logit scale. Statistical significance was defined as a two-sided *p* value < 0.05.

### Ethical considerations

The parent protocol was reviewed and approved by the Research Ethics Committee/Institutional Review Board of King Fahad Hospital Hofuf, Al-Ahsa Health Cluster, Ministry of Health, Saudi Arabia (institutional IRB number H-05-HS-065; research committee reference 40-32-2020; approval date 19 November 2020). The present study is a secondary analysis of de-identified, routinely collected data from that approved COVID-19 hospital-record cohort. No direct patient contact occurred, and confidentiality was maintained throughout. All procedures were conducted in accordance with the Declaration of Helsinki and applicable institutional requirements.

## Results

A total of 631 hospitalized patients with laboratory-confirmed COVID-19 were included. Mean age was 54.4 ± 14.4 years, and 404 of 630 patients with available sex data were male (64.1%). Liver biochemistry status could be classified for 618 patients; 366 (59.2%) had at least one abnormal parameter. Compared with patients with normal liver biochemistry, those with abnormal liver biochemistry were younger and more often male. Table 2 summarizes baseline characteristics by liver biochemistry status.

**Table 2:** Demographic and clinical characteristics by liver biochemistry status.

Variable	Overall cohort	Normal liver biochemistry (*n* = 252)	Abnormal liver biochemistry (*n* = 366)	*p* value
Age, years, mean ± SD	54.4 ± 14.4 (*n* = 631)	56.7 ± 13.5 (*n* = 252)	52.8 ± 14.8 (*n* = 366)	0.001
Male sex, *n*/*N* (%)	404/630 (64.1)	126/252 (50.0)	272/366 (74.3)	< 0.001
Diabetes mellitus, *n*/*N* (%)	205/393 (52.2)	96/157 (61.1)	103/227 (45.4)	0.003
Hypertension, *n/N* (%)	168/392 (42.9)	82/156 (52.6)	79/227 (34.8)	0.001
Chronic kidney disease, *n/N* (%)	25/391 (6.4)	9/155 (5.8)	16/225 (7.1)	0.769

The overall cohort comprised *N* = 631; liver biochemistry status was available for 618 patients (normal, *n* = 252; abnormal, *n* = 366). Values are mean ± SD or n/*N* (%). Denominators vary because analyses used available data. *p* values compare the normal and abnormal liver biochemistry groups using the tests specified in the Methods.

Liver biochemical abnormalities were frequent. ALT elevation occurred in 283 of 615 patients (46.0%), AST elevation in 281 of 617 (45.5%), and hyperbilirubinemia in 47 of 615 (7.6%). Overall, 366 of 618 patients (59.2%) met the composite definition of abnormal liver biochemistry (Table 3).

**Table 3:** Prevalence of abnormal liver biochemistry.

Liver parameter and threshold	Patients assessed, *n*	Abnormal, n/*N* (%)
ALT >40 U/L	615	283/615 (46.0)
AST >40 U/L	617	281/617 (45.5)
Total bilirubin >21 μmol/L	615	47/615 (7.6)
Any abnormal liver biochemistry	618	366/618 (59.2)

### Distribution of inflammatory markers and liver biochemistry

Table 4 summarizes the distribution of the inflammatory markers and liver biochemistry parameters. Inflammatory markers showed right-skewed distributions with wide IQRs, reflecting marked inter-individual variability. Ferritin levels were particularly dispersed, suggesting that a subgroup of patients had substantial inflammatory activation. Median ALT and AST values approached the upper limit of normal, while total bilirubin values were generally within normal limits for most patients.

**Table 4:** Distribution of inflammatory markers and liver biochemical tests.

Variable (unit)	Available *n*	Median	IQR (Q1–Q3)
CRP (mg/L)	107	9.8	4.8–14.5
Ferritin (ng/mL)	401	699.0	332.5–1429.8
D-dimer (mg/L FEU)	109	1.3	0.7–2.5
ALT (U/L)	615	38.0	24.5–59.0
AST (U/L)	617	37.0	24.0–59.0
Total bilirubin (μmol/L)	615	9.1	6.7–13.3

Values are medians and interquartile ranges. ALT, alanine aminotransferase; AST, aspartate aminotransferase; CRP, C-reactive protein; FEU, fibrinogen-equivalent units; IQR, interquartile range.

Table 5 compares inflammatory-marker distributions between patients with normal and abnormal liver biochemistry. Ferritin was significantly higher in the abnormal group. CRP and D-dimer did not differ significantly; these null findings should be interpreted cautiously because far fewer patients had CRP or D-dimer measured and test ordering was likely selective.

**Table 5:** Inflammatory markers by liver biochemistry status.

Marker (unit)	Total *n*	Normal liver biochemistry, median (IQR); *n*	Abnormal liver biochemistry, median (IQR); *n*	*p* value
CRP (mg/L)	107	8.9 (4.2–13.7); *n* = 44	10.6 (5.1–15.4); *n* = 63	0.196
Ferritin (ng/mL)	401	480.7 (174.3–953.0); *n* = 164	833.2 (499.5–1764.0); *n* = 237	< 0.001
D-dimer (mg/L FEU)	109	1.4 (0.6–2.3); *n* = 44	1.3 (0.8–2.6); *n* = 65	0.864

Values are median (IQR), with the available group-s^*^PWERpecific n shown in each cell. *p* values are from Mann–Whitney *U* tests. CRP, C-reactive protein; FEU, fibrinogen-equivalent units; IQR, interquartile range.

### Correlation between inflammatory markers and liver parameters

Table 6 presents Spearman correlations between inflammatory markers and individual liver biochemical tests. Ferritin correlated positively with ALT, AST, and total bilirubin. CRP showed a modest positive correlation with AST but not with ALT or bilirubin. D-dimer showed a weak inverse correlation with ALT and no significant correlation with AST or bilirubin. These associations do not establish causal pathways.

**Table 6:** Correlations between inflammatory markers and liver biochemical tests.

Marker	Available *n*	ALT, ρ (*p* value)	AST, ρ (*p* value)	Total bilirubin, ρ (*p* value)
CRP	107	0.13 (0.182)	0.27 (0.005)	0.04 (0.682)
Ferritin	401	0.28 (< 0.001)	0.35 (< 0.001)	0.36 (< 0.001)
D-dimer	109	−0.21 (0.028)	−0.11 (0.255)	0.05 (0.606)

Correlation cells report Spearman's ρ (*p* value); the dedicated column gives the marker-specific available-case sample size. ALT, alanine aminotransferase; AST, aspartate aminotransferase; CRP, C-reactive protein.

Table 7 presents marker-specific multivariable logistic regression models for abnormal liver biochemistry. After adjustment for age, sex, diabetes mellitus, hypertension, and chronic kidney disease, ferritin was associated with higher odds of abnormal liver biochemistry. CRP and D-dimer were not statistically significant, but their estimates were imprecise and should be interpreted cautiously because their model samples were substantially smaller.

**Table 7:** Multivariable logistic regression models for abnormal liver biochemistry.

Marker (log10-transformed)	Model *n*	aOR	95% CI	*p* value
CRP (mg/L)	107	1.65	0.73–3.72	0.228
Ferritin (ng/mL)	401	3.59	2.19–5.90	< 0.001
D-dimer (mg/L FEU)	109	1.61	0.67–3.85	0.290

Each model was adjusted for age, sex, diabetes mellitus, hypertension, and chronic kidney disease. Markers were log10-transformed; aORs therefore represent the change in odds per ten-fold increase. Model n is the marker-specific analytic sample. aOR, adjusted odds ratio; CI, confidence interval; CRP, C-reactive protein.

## Discussion

In this hospital-based cross-sectional study of patients admitted with COVID-19, abnormal liver biochemistry was common, affecting nearly three-fifths of the cohort. Among the inflammatory markers examined, ferritin demonstrated the most consistent association with hepatic biochemical abnormalities, including higher levels among patients with abnormal liver biochemistry, positive correlations with transaminases and bilirubin, and an adjusted association in multivariable analysis. In contrast, CRP and D-dimer did not show statistically significant adjusted associations, although these findings require cautious interpretation because of substantial missingness and smaller sample sizes for these markers.

The 59.2% prevalence of abnormal liver biochemistry lies within the broad range reported across COVID-19 studies, in which estimates vary substantially according to case severity, sampling time, and laboratory thresholds (4, 20). The predominance of ALT and AST abnormalities over bilirubin elevation is also consistent with Saudi and international cohorts (14, 21, 22, 24). Because AST is not liver-specific, part of the observed signal may reflect muscle injury or systemic illness rather than isolated hepatocellular damage.

Ferritin was the marker most consistently associated with abnormal liver biochemistry in this cohort. This pattern accords with reports that ferritin and other inflammatory markers rise alongside aminotransferases in hospitalized patients with COVID-19 (13, 14, 21). It is also compatible with meta-analytic evidence identifying ferritin as a marker of severe or fatal COVID-19 (11). Nevertheless, the present association should not be interpreted as predictive or causal: ferritin is influenced by systemic inflammation, iron regulation, liver disease, renal dysfunction, and other comorbid processes.

Several mechanisms could plausibly explain the ferritin–liver biochemistry association. Cytokine-mediated macrophage activation may elevate circulating ferritin while contributing to multiorgan inflammation (10, 15, 16). Experimental and clinical evidence also links ferritin and iron regulation to oxidative stress and hepatic injury (17, 18). These pathways are biologically coherent with the observed correlations, but the cross-sectional design cannot determine whether ferritin preceded, followed, or merely accompanied the biochemical abnormalities.

The composite outcome increased sensitivity for detecting commonly available biochemical abnormalities but combined measures with different tissue specificity and pathophysiology. The ALT, AST, and bilirubin thresholds were consistent with definitions used in prior COVID-19 liver research (24). However, AST may rise with muscle injury or generalized systemic illness. The endpoint should therefore be interpreted as abnormal liver biochemistry, not definitive liver-specific injury. Future studies should distinguish hepatocellular and cholestatic patterns and include alkaline phosphatase, gamma-glutamyl transferase, albumin, international normalized ratio, and imaging where available.

CRP showed only a modest correlation with AST and was not independently associated with the composite outcome. Other cohorts have found higher CRP, ferritin, and D-dimer among patients with abnormal liver tests (13, 14, 21), so the weaker CRP result here may reflect measurement timing, selective test ordering, or limited power rather than absence of systemic inflammation. CRP kinetics and its lack of liver specificity also limit interpretation (12, 25). D-dimer was likewise not independently associated. Although D-dimer is an established marker of COVID-19-associated coagulation activation and adverse prognosis (19, 26), that prognostic role does not necessarily imply a direct association with the composite liver-biochemistry outcome.

### Implications of the study

The findings have clinical and research implications. First, the high prevalence of abnormal liver biochemistry among hospitalized COVID-19 patients supports routine monitoring of liver biochemical tests during inpatient care, particularly when patients have severe systemic inflammation or are receiving potentially hepatotoxic medications.

Second, ferritin may be a clinically informative marker for identifying patients more likely to have concurrent abnormal liver biochemistry. This does not establish ferritin as a predictive or causal marker, but it suggests that ferritin could be considered alongside liver biochemistry as part of broader clinical assessment in hospitalized patients.

Third, the findings support further investigation of immune-mediated pathways of liver involvement in COVID-19. Prospective multicenter studies with standardized laboratory measurement, longitudinal sampling, and more complete adjustment for COVID-19 severity, medications, metabolic risk factors, and chronic liver disease are needed to clarify temporality and clinical usefulness.

### Limitations of the study

Several limitations should be considered. First, the cross-sectional design precludes causal inference and does not establish the temporal sequence between inflammatory-marker elevation and abnormal liver biochemistry. Second, inflammatory markers were not measured uniformly, particularly CRP and D-dimer; clinically selective ordering creates non-random missingness and may have reduced power. Third, residual confounding is likely because COVID-19 severity, intensive care admission, oxygen requirement, body mass index, hepatotoxic medications, alcohol use, viral hepatitis, and detailed pre-existing liver conditions were not consistently available. Fourth, the composite outcome cannot fully distinguish liver-specific injury from systemic illness, particularly because it includes AST. Fifth, this secondary analysis was limited to variables available in the parent cohort. Finally, the single-center data were collected during August–October 2020, before vaccination and later variants and treatment protocols, limiting generalizability to other settings and contemporary COVID-19 care.

## Conclusion

Abnormal liver biochemistry was common among hospitalized patients with COVID-19, with a predominance of hepatocellular enzyme abnormalities. Ferritin showed the most consistent association with abnormal liver biochemistry after adjustment for age, sex, and major comorbidities in the primary model. CRP and D-dimer were not independently associated, although interpretation is limited by greater missingness and smaller available samples for these markers. Prospective multicenter studies with standardized laboratory assessment and more comprehensive adjustment for disease severity and hepatic risk factors are needed to confirm these findings and clarify their clinical utility.

## Data Availability

The raw data supporting the conclusions of this article will be made available by the authors, without undue reservation.
